# Non-Invasive Evaluation of Cystic Fibrosis Related Liver Disease in Adults with ARFI, Transient Elastography and Different Fibrosis Scores

**DOI:** 10.1371/journal.pone.0042139

**Published:** 2012-07-25

**Authors:** Thomas Karlas, Marie Neuschulz, Annett Oltmanns, Andrea Güttler, David Petroff, Hubert Wirtz, Jochen G. Mainz, Joachim Mössner, Thomas Berg, Michael Tröltzsch, Volker Keim, Johannes Wiegand

**Affiliations:** 1 University Hospital Leipzig, Department of Internal Medicine, Dermatology and Neurology, Medical Clinic for Gastroenterology and Rheumatology, Leipzig, Germany; 2 University Hospital Leipzig, Department of Internal Medicine, Dermatology and Neurology, Division of Pulmonary Medicine, Leipzig, Germany; 3 University of Leipzig, Coordination Center for Clinical Trials, Leipzig, Germany; 4 University Hospital Jena, Department of Pediatrics, Jena, Germany; 5 University Hospital Leipzig, Interdisciplinary Ultrasound Unit, Leipzig, Germany; University of Tübingen, Germany

## Abstract

**Background:**

Cystic fibrosis-related liver disease (CFLD) is present in up to 30% of cystic fibrosis patients and can result in progressive liver failure. Diagnosis of CFLD is challenging. Non-invasive methods for staging of liver fibrosis display an interesting diagnostic approach for CFLD detection.

**Aim:**

We evaluated transient elastography (TE), acoustic radiation force impulse imaging (ARFI), and fibrosis indices for CFLD detection.

**Methods:**

TE and ARFI were performed in 55 adult CF patients. In addition, AST/Platelets-Ratio-Index (APRI), and Forns' score were calculated. Healthy probands and patients with alcoholic liver cirrhosis served as controls.

**Results:**

Fourteen CF patients met CFLD criteria, six had liver cirrhosis. Elastography acquisition was successful in >89% of cases. Non-cirrhotic CFLD individuals showed elastography values similar to CF patients without liver involvement. Cases with liver cirrhosis differed significantly from other CFLD patients (ARFI: 1.49 vs. 1.13 m/s; p = 0.031; TE: 7.95 vs. 4.16 kPa; p = 0.020) and had significantly lower results than individuals with alcoholic liver cirrhosis (ARFI: 1.49 vs. 2.99 m/s; p = 0.002). APRI showed the best diagnostic performance for CFLD detection (AUROC 0.815; sensitivity 85.7%, specificity 70.7%).

**Conclusions:**

ARFI, TE, and laboratory based fibrosis indices correlate with each other and reliably detect CFLD related liver cirrhosis in adult CF patients. CF specific cut-off values for cirrhosis in adults are lower than in alcoholic cirrhosis.

## Introduction

Cystic fibrosis (CF) is the most common lethal genetic disease in populations of European descent. Cystic fibrosis-related liver disease (CFLD) is caused by biliary hyperviscosity which results in bile duct obstruction and is present in up to one third of CF patients. Five to 10% of patients develop liver cirrhosis and may require liver transplantation [1]. Diagnosis of CFLD is challenging because its clinical presentation ranges from simple steatosis to multi-lobular biliary cirrhosis with portal hypertension [2], [3]. Non-invasive scoring systems based on liver sonomorphology [4], [5] and biochemical parameters [6], [7] have been developed. However, in many studies these diagnostic criteria were modified resulting in heterogeneous data of CFLD prevalence [1]. The focal pattern of liver involvement results in disaccordance between clinical findings and histological staging and a high proportion of sampling errors [8]. Therefore, although liver biopsy may help to identify patients at risk for development of clinically significant liver disease [9] it is not a standard procedure in CF [1].

In the last decade, ultrasound-based elastography methods for non-invasive assessment of chronic liver diseases have been developed [10], [11]. First results of transient elastography (Fibroscan®, TE) in cystic fibrosis revealed an elevated liver stiffness in patients with CFLD classified according to pathologic ultrasound criteria [12] and biochemical or clinical definitions [13]. In a case series, TE could reliably identify children with CFLD and portal hypertension [14]. First data with the recently developed acoustic radiation force impulse imaging (ARFI) technique revealed a high accuracy for the detection of liver cirrhosis in pediatric CF patients with portal hypertension [15].

However, liver elastography has not been systematically evaluated in adult patients, and a direct comparison of TE and ARFI is missing so far. Thus, the aim of the present study was to evaluate both methods simultaneously in a cohort of adult CF patients.

## Materials and Methods

### Ethical statement

The study was performed in strict accordance with the ethical guidelines of the Helsinki Declaration and was approved by the Leipzig University ethics committee (registration number 091-10-19042010). All study participants provided written informed consent.

### Patients and controls

Adult CF patients were prospectively investigated at presentation to the pulmonary outpatient clinic for clinical routine examinations. Patients with pregnancy, age <18 years, and liver transplantation were not included. Patients underwent conventional upper abdomen ultrasound evaluation, elastography and blood tests (alanine aminotransferase (ALT), aspartate aminotransferase (AST), alkaline phosphatase (AP), bilirubin, gamma-glutamyltransferase (GGT), blood count, INR, albumin, creatinine, and cholesterol) at the same day. Fasting for at least three hours was required prior to examination, however exceptions were permitted when clinically required.

Previous ultrasound reports, recent pulmonary function tests (time span <6 months), and results of previous routine blood tests were collected from clinical records.

Cystic fibrosis-related liver disease was defined if at least 2 of the following conditions were present on at least 2 consecutive examinations spanning a 1-year period [6], [7]: (1) Ultrasound confirmed hepatomegaly; (2) elevated serum liver enzyme levels of ALT, AST, AP, or GGT; (3) ultrasound abnormalities other than hepatomegaly (i.e., increased, heterogeneous echogenicity, nodularity, irregular margins, splenomegaly). An ultrasonographic pattern of simple liver steatosis did not represent a diagnostic criterion. In case of distinct ultrasonographic signs of liver cirrhosis (i.e. coarse nodularity, presence of portal hypertension and rarefication of peripheral portal veins) and clinical signs (e.g. esophageal varices, splenomegaly) of liver cirrhosis CFLD patients were classified as cirrhotics.

ARFI results were compared with a control group of 50 healthy volunteers which has already been described in detail before [16]. Ten patients with alcoholic liver cirrhosis served as positive controls.

### Acoustic radiation force impulse (ARFI) elastography

Liver stiffness was measured by ARFI technology (Acuson S2000; Siemens Medical Solutions, Mountain View, California, USA; Software Version 350.1.050.36) using a convex probe (4C1, Siemens Healthcare).

Patients were examined in a supine position with the right arm elevated above the head in a resting respiratory position if tolerable (short breath-hold without deep inspiration). The measurement depth from the transducer surface was between 20 and 55 mm. The examiner aimed for a measuring angle close to 0° (region of interest position in the center of the transducer surface).

Measurements were performed at two different sites in an area of homogenous tissue: i) right liver lobe through the intercostal space and ii) left liver lobe in the epigastric region in the median line. Measurements were performed by experienced operators (TK, VK, MN, MT, JW).

10 valid measurements at each site were required. The ARFI success rate was calculated as the ratio between the valid and the total number of measurements. The median value of each measuring site was used for further analysis. In case of a success rate below 60% the result was regarded as invalid and excluded from further analysis [16], [17].

### Transient elastography (TE)

For transient elastography (TE; Fibroscan®, Echosens, Paris, France; Software Version 1.40) all subjects were examined in a supine position immediately after ARFI measurement. TE was performed in a right intercostal space in resting respiratory position. 10 valid measurements were taken according to the manufacturer's recommendation (M probe). Measurements were performed by experienced operators (TK, VK, MN, MT). Patients with an interquartile range (IQR)>median value/3 or a success rate below 60% were considered as invalid and excluded from further analysis.

### Non-invasive fibrosis indices

In addition to ultrasound examination, liver fibrosis was investigated by the two non-invasive fibrosis indices AST/Platelets-Ratio-Index (APRI) and Forns' score [18], [19]. Forns' score was calculated according to the formula [19]:


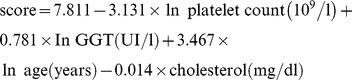


### Statistical Analysis

Ordinal and nominal data were collected in a Microsoft® Excel file. Statistical analyses were conducted by using MedCalc® 11.4 (MedCalc Software, Belgium) and PASS 11.0.2 (NCSS, UT; USA). Clinical and laboratory data were expressed as median ± range and mean ± standard deviation (SD) where appropriate. Elastography results are presented as boxplots.

Fisher's exact test and chi-square test were used to test for association of variables. Nonparametric tests were chosen to compare median values of two independent samples (Mann-Whitney U test) or groups (Kruskal-Wallis test). Correlations between variables were examined using the Pearson's correlation coefficient. Diagnostic performance of elastography methods and serologic fibrosis indices were evaluated using receiver operating characteristic curves. The probabilities of a true-positive (sensitivity) and a true-negative (specificity) assessment with selected cut-off values were determined, and the area under the receiver operating characteristics curve (AUROC) was calculated [20]. All tests were two-sided. A p-value<0.05 indicated a significant difference or correlation.

## Results

### Baseline characteristics of the total study cohort

55 adult CF patients were prospectively included in the study from April to December 2010 (table 1). Fourteen of 55 patients (25.5%) met the criteria for CFLD. There were no significant differences between CFLD patients and those without liver involvement concerning age, body mass index, duration of CF, age of initial CF diagnosis, and results of pulmonary function tests. Treatment with ursodeoxycholic acid (UDCA) was more frequent in the CFLD group (93% vs. 54%, p = 0.010). Only one patient of the CFLD group (7%), but seven cases without CFLD (17%) had undergone lung transplantation (p = 0.664).

**Table 1:** Baseline characteristics of the study cohort.

	Cystic fibrosis patients			CFLD subgroups		Healthy controls	Alcoholic cirrhosis
	Total study cohort	Without CFLD	With CFLD	Without cirrhosis	With cirrhosis		
Gender (n)							
Male/female	31/24	24/17	7/7	4/4	3/3	21/29	8/2
Age (years)*	31.9±8.8	32.9±9.0	29.0±8.0	29.6±7.8	28.3±8.9	27.8±4.0	57.5±5.9
BMI (kg/m^2^)*	21.5±3.8	21.7±4.1	20.8±3.0	20.9±3.0	20.6±3.2	22.1±1.9	26.0±5.1
Time (years) since initial CF diagnosis*	26.6±10.1	26.2±10.9	27.8±7.3	28.4±7.6	27.0±7.5	/	/
Age (years) at CF diagnosis*	5.2±10.7	6.6±12.0	1.1±1.2	1.3±1.0	1.0±1.5	/	/
CFTR-genotype ΔF508/other (n)	37/18	27/12	12/4	5/3	5/1		
Lung transplantation (n)	8	7	1	1	0	/	/
Ursodeoxycholic acid treatment (n)	35	22	13	7	6	/	/
Diabetes mellitus (n)	18	11	7	5	2	/	/
FVC (l)*	3.2±1.2	3.2±1.2	3.2±1.3	3.1±1.3	3.3±1.5	/	/
FEV1 (l/s)*	2.3±1.0	2.3±0.9	2.4±1.2	2.3±1.1	2.4±1.4	/	/
FEV1 in % of VC	71.2±13.1	70.6±13.5	72.8±12.1	74.8±11.2	70.5±13.7		
TLC (l)*	6.1±1.2	6.2±1.1	5.9±1.4	6.2±1.2	5.7±1.7	/	/
RV (l)*	2.8±0.9	2.9±0.9	2.5±0.9	2.8±1.0	2.2±0.6	/	/
*in % of predicted value*							
- FVC (%)	73.8±23.1	72.5±21.6	77.4±27.7	74.5±30.4	80.9±26.6		
- FEV1 (%)	62.3±24.8	60.5±22.3	67.5±31.5	65.8±32.4	69.9±33.3		
- TLC (%)	102.0±12.2	101.0±11.8	105.0±13.5	105.2±11.9	104.9±16.5		
- RV (%)	172.7±54.3	173.8±52.6	169.5±61.1	178.0±72.6	159.7±49.2		
Platelets (10^9^/l)*	308.1±135.9	341.0±131.0	211.7±102.5	269.1±80.0	135.2±77.7	/	164.9±87.2
ALT (µkat/l)*	0.5±0.2	0.4±0.2	0.6±0.4	0.4±0.2	0.8±0.4	/	0.5±0.2
AST (µkat/l)*	0.5±0.2	0.5±0.1	0.6±0.2	0.5±0.1	0.7±0.3	/	0.9±0.4
AP (µkat/l)*	2.00±1.37	1.63±0.59	3.08±2.25	1.98±0.51	4.56±2.86	/	1.8±0.8
GGT (µkat/l)*	0.69±1.11	0.55±0.81	1.12±1.67	0.44±0.18	2.04±2.34	/	2.7±2.3
Bilirubin (µmol/l)*	8.1±7.2	6.6±3.0	12.5±12.3	7.3±4.8	19.4±16.2	/	23.1±15.0
Albumin (g/l)*	43.8±4.3	43.9±4.5	43.6±4.1	43.2±4.3	44.0±4.2	/	35.1±12.3
INR*	1.0±0.1	1.0±0.1	1.1±0.1	1.0±0.1	1.1±0.1	/	1.3±0.2

* mean ± standard deviation.

CFLD – cystic fibrosis-related liver disease; FVC – functional vital capacity; FEV1 – forced expiratory volume in 1 second; VC – vital capacity, TLC – total lung volume; RV – residual lung volume.

In the ARFI control group, healthy subjects were significantly younger than the CF individuals (p = 0.012). However, the age difference was moderate (12% of median CF patient age) and there was no correlation between age and liver stiffness.

The group of patients with alcoholic liver cirrhosis was significantly older (p = 0.002) and had a higher body mass index than CF-cases with cirrhosis (p = 0.025). None of the individuals presented with either acute hepatitis (aminotransferase levels >2× upper limit of normal) or ascites.

### Baseline characteristics of CFLD-patients

Six of the CFLD patients had distinct clinical and sonographical signs of advanced liver disease, three of them showed collateral circulation of the portal vein. These six cases were classified as CFLD cirrhosis (table 1).

CF patients with cirrhosis showed higher values of alkaline phosphatase (p = 0.028), bilirubin (p = 0.039) and a significantly lower platelet count than CFLD cases without cirrhosis (135 vs. 269×10^9^/l, p = 0.010). There were no significant differences concerning age, body mass index, time since and age at CF diagnosis, and pulmonary function. The distribution of ΔF508 CFTR-genotype, treatment with ursodeoxycholic acid, and the presence of diabetes mellitus did not vary significantly.

### Practicability of ARFI, transient elastography, and fibrosis indices

ARFI-acquisition was completely successful in 53 cases (96%). In two cases without CFLD the success rate was <60% for one liver lobe.

TE measurements were invalid in six cases without CFLD (one patient: success rate <60%; five patients: IQR exceeding the third of the median value) resulting in a rate of valid measurements of 89. Rates of valid results between ARFI and TE were not significantly different (p = 0.271).

APRI score and Forns index could be calculated in all CF patients.

### Results of ARFI, transient elastography, and fibrosis indices

**Figure 1:**
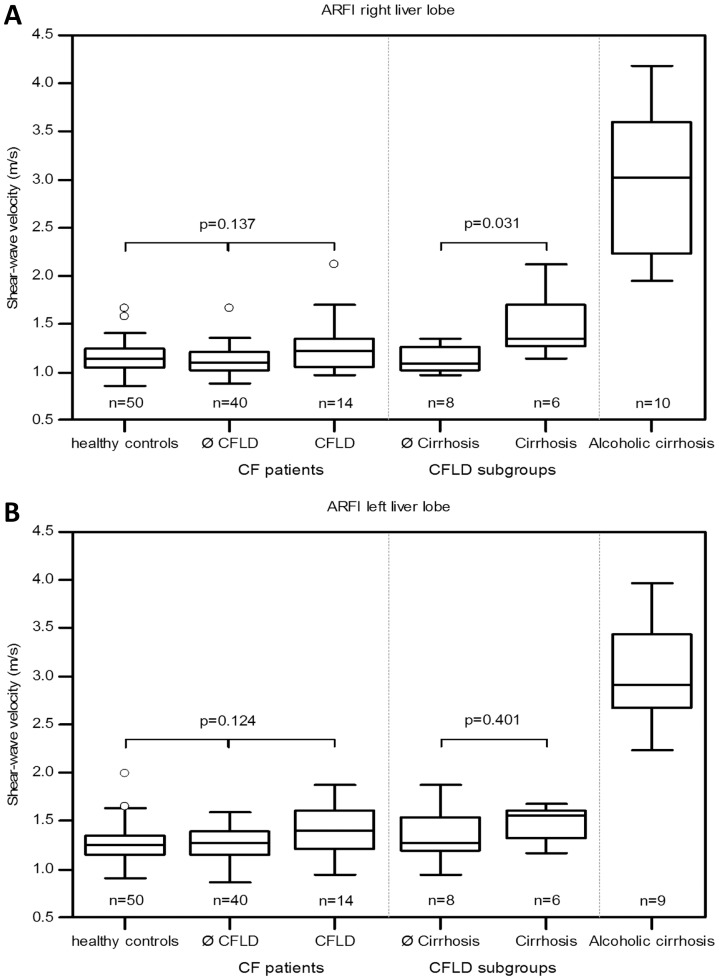
ARFI results in adult cystic fibrosis (CF) patients and controls. Results of shear-wave velocity measurement in the liver tissue are shown as boxplots (median, 25%- and 75% quartile, maximum and minimum, outliers) according to the site of measurement. Shear-wave velocity is not increased in CF-related liver disease (CFLD) patients compared to healthy controls and CF patients without liver involvement. ARFI measurement in the right liver lobe (A) can detect patients with cirrhosis in the CFLD group. Patients with alcoholic liver cirrhosis have significantly higher shear-wave velocity values than CF patients with cirrhosis (p = 0.002).

**Figure 2:**
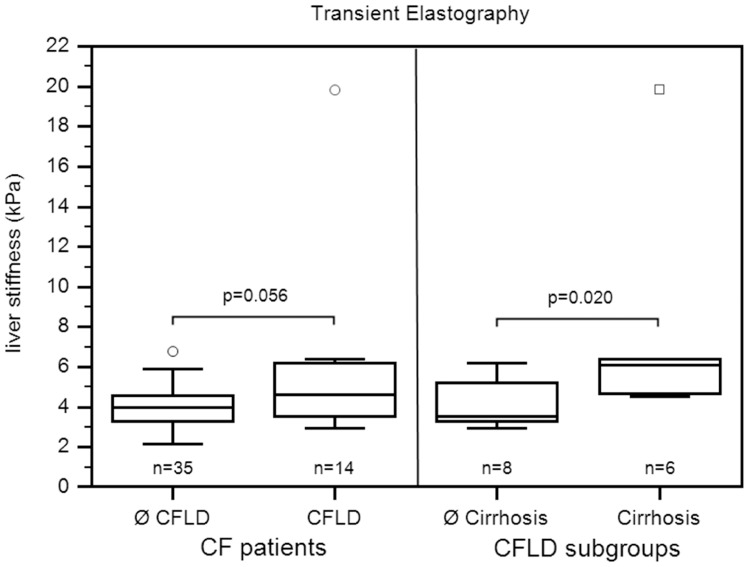
Transient elastography results in cystic fibrosis (CF) patients. Results of liver stiffness measurement are shown as boxplots (median, 25%- and 75% quartile, maximum and minimum, outliers). Liver stiffness is not increased in CF-related liver disease (CFLD) patients compared to CF patients without liver involvement. CFLD-patients with liver cirrhosis show a significant increase of liver stiffness.

Results of elastography measurements and serologic fibrosis indices are shown in table 2. Individuals with CFLD had ARFI and TE values similar to CF patients without liver involvement in both liver lobes. ARFI results did not differ between both subgroups and healthy controls (figures 1 and 2). Direct comparison of ARFI results without Bonferroni correction for the negative control group revealed a slight difference of shear-wave velocities in the right liver lobe between the CFLD patients and the group without CFLD (1.28±0.31 vs. 1.12±0.15 m/s; p = 0.044; Mann-Whitney U test).

**Table 2:** Results of elastography and fibrosis scores.

	Cystic fibrosis patients			CFLD subgroups		Healthy controls	Alcoholic cirrhosis
	Total study cohort	Without CFLD	With CFLD	Without cirrhosis	With cirrhosis		
	n = 55	n = 41	n = 14	n = 8	n = 6	n = 50	n = 10
**Elastography** *							
R-ARFI (m/s)	1.16±0.21	1.12±0.15	1.28±0.31	1.13±0.15#	1.49±0.36# ^,^ †	1.15±0.17	2.99±0.77†
Success rate	0.95±0.13	0.94±0.14	0.96±0.09	0.94±0.11	0.98±0.04	0.95±0.09	0.91±0.09
valid/invalid (n)	54/1	40/1	14/0	8/0	6/0	50/0	10/0
L-ARFI (m/s)	1.29±0.21	1.25±0.18	1.41±0.25	1.35±0.29	1.48±0.20†	1.28±0.19	3.05±0.54†
Success rate	0.92±0.16	0.91±0.17	0.95±0.09	0.96±0.06	0.95±0.12	0.93±0.09	0.88±0.20
valid/invalid (n)	54/1	40/1	14/0	8/0	6/0	50/0	9/1
TE (kPa)	4.5±2.5	4.0±1.1	5.8±4.2	4.2±1.3#	8.0±5.9#	/	/
Success rate	0.94±0.10	0.94±0.10	0.96±0.08	92.8±8.9	100	/	/
valid/invalid (n)	49/6	35/6	14/0	8/0	6/0	/	/
**Fibrosis Indices** *							
APRI	0.314±0.395	0.207±0.099	0.630±0.687	0.28±0.12#	1.09±0.87#	/	/
Forns	2.471±2.064	1.933±1.627	4.045±2.444	2.66±1.17#	5.89±2.54#	/	/

* results shown as mean ± standard deviation.

# significant difference compared to other subgroup (p-value<0.03).

† significant difference compared to positive control group (p-value = 0.002).

CFLD – cystic fibrosis-related liver disease; TE – transient elastography, R-ARFI – right liver lobe ARFI; L-ARFI – left liver lobe ARFI; APRI – AST/Platelets-Ratio-Index.

Both ARFI and TE did not reveal a significant difference between CF patients without liver involvement and non-cirrhotic CFLD cases (n = 8): 1.12±0.15 vs. 1.13±0.15 m/s (right liver lobe ARFI, p = 0.83) and 4.0±1.1 vs. 4.2±1.3 kPa (TE, p = 0.94), respectively (table 2). However, CFLD cases with liver cirrhosis differed significantly from CFLD patients without cirrhosis in right liver lobe ARFI (1.49±0.36 vs. 1.13±0.15 m/s; p = 0.031) and TE (7.95±5.88 vs. 4.16±1.28 kPa; p = 0.020). Patients with alcoholic liver cirrhosis had significantly increased values of shear-wave velocity in both right and left liver lobe compared to CF patients with cirrhosis (2.99±0.77 vs. 1.49±0.36 m/s and 3.05±0.54 vs. 1.48±0.20 m/s; p = 0.002, respectively).

TE results were highly correlated with median ARFI values of the right liver lobe in all patients (rho = 0.762; p<0.001). ARFI values of the left liver lobe showed good correlation with right liver lobe ARFI (rho = 0.311; p<0.001) and with TE (rho = 0.333; p<0.021). All elastography methods displayed a significant correlation with APRI and Forns indices (rho>0.4; p<0.01).

The diagnostic performance for CFLD and cirrhosis detection was comparable between elastography methods and the serological fibrosis indices (table 3). Sensitivity of ARFI and TE for CFLD detection was low (50.0% and 42.9%, respectively). However, specificity was 92.5% and 97.1%. Among all methods, the APRI score showed the highest area under the receiving operator characteristics curve values for CFLD detection with 85.7% sensitivity and 70.7% specificity. In CFLD subgroups, diagnostic accuracy of ARFI (right liver lobe), TE, APRI and Forns index for detection of cirrhosis was almost equal (p<0.03, respectively) (table 3).

**Table 3:** Area Under Receiving Operating Characteristics Curve (95% CI), cut-off value, sensitivity and specificity for the detection of adult cystic fibrosis-related liver disease (CFLD) and cirrhosis.

	Elastography			Fibrosis Indices	
	ARFI (right lobe)	ARFI (left lobe)	TE	APRI	FORNS
**No CFLD vs. CFLD (n)**	40/14	40/14	35/14	41/14	41/14
AUROC*	0.682 [0.541; 0.802]	0.672 [0.531; 0.794]	0.677 [0.528; 0.803]	0.815 [0.688; 0.907]	0.786 [0.654; 0.885]
Cut-Off	1.28 m/s	1.43 m/s	5.9 kPa	0.231	2.154
Sensitivity	42.9%	50.0%	42.9%	85.7%	92.9%
Specificity	92.5%	90.0%	97.1%	70.7%	61.0%
**Subgroup analysis**					
CFLD-no cirrhosis vs. CFLD-cirrhosis (n)	8/6	8/6	8/6	8/6	8/6
AUROC*	0.854 [0.568; 0.981]	0.635 [0.345; 0.868]	0.875 [0.593; 0.988]	0.875 [0.593; 0.988]	0.854 [0.568; 0.981]
Cut-Off	1.13 m/s	1.47 m/s	4.4 kPa	0.344	4.059
Sensitivity	100%	66.7%	100%	83.3%	66.7%
Specificity	62.5%	75.0%	75.0%	87.5%	100%

* 95% confidence interval.

CFLD – cystic fibrosis-related liver disease; AUROC area under receiver operating characteristics curve.

## Discussion

This is the first prospective study evaluating TE and ARFI simultaneously for the detection of CFLD in a cohort of adult CF patients presenting with baseline characteristics comparable to previously reported studies [1], [7].

ARFI and TE could be applied with a success rate>90% and correlated highly between each other. However, TE had a higher rate of invalid measurements than ARFI in patients without CFLD (15% vs. 2%) which is in line with previously reported TE failure rates [12]. This phenomenon may be partially explained by CF specific conditions like hypertrophic intercostal muscles or by difficulties to tolerate necessary standardized examination procedures (i.e. supine position, short respiratory holds) during the examination in case of CF-related respiratory impairment [16].

Both TE and ARFI could not significantly discriminate between non-cirrhotic CFLD patients and CF cases without liver disease although a gradual increase would have been expected. This observation should be interpreted very cautiously due to the limited case number in the non-cirrhotic CFLD subgroup. However, TE and ARFI (right liver lobe) distinguished between liver cirrhosis and earlier stages of liver disease in CFLD patients.

Previous studies suggested a potential diagnostic benefit of left liver lobe ARFI in healthy controls which seems to diminish in advanced liver disease [16], [21]. However, in our CF cohort left liver lobe ARFI failed to discriminate between CFLD and non CFLD patients and was not able to detect liver cirrhosis in CF patients. Thus, it remains unclear whether left liver lobe ARFI provides an additional diagnostic benefit.

Elastography values in CF patients with liver cirrhosis were significantly lower than in alcoholic cirrhosis and differed from reported cut-off values for liver cirrhosis in chronic hepatitis B and C [11], [16], [22]. Interestingly, in relation to healthy individuals shear-wave velocity in cirrhotic CF patients were only marginally increased (1.49±0.36 vs. 1.15±0.17 m/s; p = 0.007). However, even this subtile difference of liver stiffness seems to be typical for adult CF patients and allows reliable application of ARFI and TE. It strengthens the assumption that elastography values depend on the etiology of liver disease [23].

There is a set of possible explanations for our findings:

- Up to two thirds of pediatric and adolescent CF patients show an ultrasound pattern of fatty liver disease [3], [24], [25] while the incidence is lower in adult CF patients [26]. In our adult cohort, 12/55 (22%) individuals displayed signs of hepatic steatosis at ultrasound evaluation. A direct relation of steatosis and CFLD is questionable, and extrahepatic CF manifestations (i.e. malnutrition, essential fatty acid deficiency, insulin resistance) have been discussed as potential causes as well [3]. Fatty liver disease modulates liver stiffness and can impair reliability of elastography for the detection of fibrosis. Patients with fatty liver disease and advanced fibrosis may not be detected by TE because of a higher hepatic fat content [27]. 3/6 cases classified as CF-related cirrhosis displayed an increased echogenicity of liver parenchyma (steatosis grad I) in our investigations. Thus, the marginally elevated elastography results in our adult CF cirrhotic patients may be influenced by hepatic steatosis.
- CFLD is a known risk factor for a severe CF course in children. However, the prognostic role of CFLD in adults is less certain, and disease progression after adolescence is rare [7], [26]. Previous studies consisted of mixed patient cohorts with a large proportion of pediatric patients [12], [13] or of CF children with portal hypertension [14]. Hence, the reported increase of liver stiffness in these CFLD patients may be related to the more severe course of pediatric CFLD while our results reflect a milder degree of liver damage in adult CFLD cases.
- The use of different CFLD definitions in previous studies limits their comparability with our results [12], [13]. We defined CFLD according to Colombo's criteria [1]. Though these criteria are sensitive for persistent liver impairment, the diagnosis of CFLD is not always associated with significant liver fibrosis [9]. The absence of fibrosis in some cases may have limited accuracy of ARFI and TE for CFLD detection in our study. In addition, limited accuracy of all investigated ultrasound- and laboratory-based methods to detect liver fibrosis may be also influenced by diagnostic limitations of our reference method B-mode ultrasound. Conventional ultrasound may misclassify patients as non-cirrhotic although they are cirrhotic in reality.
- The number of recruited patients - although comparable to previous studies [12], [13] - was not sufficient to detect a significant difference of liver stiffness between CFLD patients and those without CFLD. Based on a post-hoc power calculation (alpha error 0.05, beta error 0.1), a much larger case number would have been required for reliable CFLD detection (n = 138 for TE and n = 171 for right liver lobe ARFI, respectively). In addition, our case number may have been too small to reveal correlations between known risk CFLD factors and elastography results although there was a trend of earlier age at diagnosis in CFLD patients.

Ideally, non-invasive evaluation of liver fibrosis should be compared to adequate results of liver biopsy. However, the focal nature of liver damage in CFLD is the key problem for histological assessment and limits its use as standard classification in CF [1]. If focal liver disease affects biopsy, elastography will be affected as well. The tissue volume assessed with TE and ARFI is relatively small and even B-mode ARFI image measurement control cannot exclude “acoustic biopsy sampling errors”. However, we investigated two different established elastography methods simultaneously and performed ARFI measurements in both liver lobes. There was a high correlation of measurement results and thus a sampling bias is unlikely. Moreover, we calculated APRI and Forns index and showed comparable accuracy with ARFI and TE for CFLD and cirrhosis detection. The APRI score performed best compared to all other evaluated tests in our cohort. It may be an interesting and easy to perform alternative for non-invasive assessment of CFLD in adult patients which deserves further evaluation in the future although it should be noticed that it was unable to detect liver disease in pediatric patients before [13]. This difference may be explained by age related levels of aminotransferases and platelets [28] which can result in imprecise upper limits of normal and succeeding impaired diagnostic performance of APRI.

In summary, right liver lobe ARFI and TE correlate with each other in CF patients and can reliably detect CFLD related liver cirrhosis. CF specific elastography cut-off values in adults are lower compared to liver diseases of other etiologies. A structured and prospective use of ultrasound- and laboratory based approaches to investigate CF-related liver disease may detect progression of liver fibrosis invisible by ultrasound and allow further risk stratification in adult CF patients.

## References

[1] Debray D, Kelly D, Houwen R, Strandvik B, Colombo C (2011) Best practice guidance for the diagnosis and management of cystic fibrosis-associated liver disease. J Cyst Fibros 10: S29–36. 21658639 10.1016/S1569-1993(11)60006-4

[2] Colombo C, Battezzati PM (2004) Liver involvement in cystic fibrosis: primary organ damage or innocent bystander? J Hepatol 41: 1041–1044. 15582140 10.1016/j.jhep.2004.10.002

[3] Moyer K, Balistreri W (2009) Hepatobiliary disease in patients with cystic fibrosis. Curr Opin Gastroenterol 2009 25: 272–278. 10.1097/MOG.0b013e3283298865 19381084

[4] Williams SG, Evanson JE, Barrett N, Hodson ME, Boultbee JE, et al (1995) An ultrasound scoring system for the diagnosis of liver disease in cystic fibrosis. J Hepatol 22: 513–521. 7650330 10.1016/0168-8278(95)80444-7

[5] Williams SM, Goodman R, Thomson A, McHugh K, Lindsell DRM (2002) Ultrasound evaluation of liver disease in cystic fibrosis as part of an annual assessment clinic: a 9-year review. Clin Radiol 57: 365–370. 12014933 10.1053/crad.2001.0861

[6] Sokol RJ, Durie PR (1999) Recommendations for management of liver and biliary tract disease in cystic fibrosis. Cystic Fibrosis Foundation Hepatobiliary Disease Consensus Group. J Pediatr Gastroenterol Nutr 28: S1–13. 9934970 10.1097/00005176-199900001-00001

[7] Colombo C, Battezzati PM, Crosignani A, Morabito A, Costantini D, et al (2002) Liver disease in cystic fibrosis: A prospective study on incidence, risk factors, and outcome. Hepatology 36: 1374–1382. 12447862 10.1053/jhep.2002.37136

[8] Mueller-Abt PR, Frawley KJ, Greer RM, Lewindon PJ (2008) Comparison of ultrasound and biopsy findings in children with cystic fibrosis related liver disease. J Cyst Fibros 7: 215–221. 17904429 10.1016/j.jcf.2007.08.001

[9] Lewindon PJ, Shepherd RW, Walsh MJ, Greer RM, Wiliamson R, et al (2011) Importance of hepatic fibrosis in cystic fibrosis and the predictive value of liver biopsy. Hepatology 53: 193–201. 21254170 10.1002/hep.24014

[10] Sandrin L, Fourquet B, Hasquenoph J, Yon S, Fournier C, et al (2003) Transient elastography: a new noninvasive method for assessment of hepatic fibrosis. Ultrasound Med Biol 29: 1705–1713. 14698338 10.1016/j.ultrasmedbio.2003.07.001

[11] Friedrich-Rust M, Wunder K, Kriener S, Sotoudeh F, Richter S, et al (2009) Liver fibrosis in viral hepatitis: noninvasive assessment with acoustic radiation force impulse imaging versus transient elastography. Radiology 252: 595–604. 19703889 10.1148/radiol.2523081928

[12] Menten R, Leonard A, Clapuyt P, Vincke P, Nicolae A, et al (2010) Transient elastography in patients with cystic fibrosis. Pediatr Radiol 40: 1231–1235. 20135110 10.1007/s00247-009-1531-z

[13] Witters P, Boeck Kde, Dupont L, Proesmans M, Vermeulen F, et al (2009) Non-invasive liver elastography (Fibroscan) for detection of cystic fibrosis-associated liver disease. J Cyst Fibros 8: 392–399. 19733131 10.1016/j.jcf.2009.08.001

[14] Malbrunot-Wagner AC, Bridoux L, Nousbaum JB, Riou C, Dirou A, et al (2011) Transient elastography and portal hypertension in pediatric patients with cystic fibrosis Transient elastography and cystic fibrosis. J Cyst Fibros 10: 338–342. 21550861 10.1016/j.jcf.2011.04.004

[15] Manco M, Lo Zupone C, Latini A, Lucidi V, Monti L (2011) Noninvasive assessment of cystic fibrosis-associated liver disease with acoustic radiation force impulse imaging. Hepatology 53: 1779–1780. 21351113 10.1002/hep.24245

[16] Karlas T, Pfrepper C, Wiegand J, Wittekind C, Neuschulz M, et al (2011) Acoustic radiation force impulse imaging (ARFI) for non-invasive detection of liver fibrosis: examination standards and evaluation of interlobe differences in healthy subjects and chronic liver disease. Scand J Gastroenterol 46: 1458–1467. 21916815 10.3109/00365521.2011.610004

[17] Bota S, Sporea I, Sirli R, Popescu A, Dănilă M, et al (2011) Factors that influence the correlation of acoustic radiation force impulse (ARFI) elastography with liver fibrosis. Med Ultrason 13: 135–140. 21655540

[18] Lin ZH, Xin YN, Dong QJ, Wang Q, Jiang XJ, et al (2011) Performance of the aspartate aminotransferase-to-platelet ratio index for the staging of hepatitis C-related fibrosis: an updated meta-analysis. Hepatology 53: 726–736. 21319189 10.1002/hep.24105

[19] Forns X, Ampurdanès S, Llovet JM, Aponte J, Quinto L, et al (2002) Identification of chronic hepatitis C patients without hepatic fibrosis by a simple predictive model. Hepatology 36: 986–992. 12297848 10.1053/jhep.2002.36128

[20] DeLong ER, DeLong DM, Clarke-Pearson DL (1988) Comparing the areas under two or more correlated receiver operating characteristic curves: a nonparametric approach. Biometrics 44: 837–845. 3203132

[21] D'Onofrio M, Gallotti A, Mucelli RP (2010) Tissue quantification with acoustic radiation force impulse imaging: Measurement repeatability and normal values in the healthy liver. AJR Am J Roentgenol 195: 132–136. 20566806 10.2214/AJR.09.3923

[22] Sporea I, Sirli RL, Deleanu A, Popescu A, Focsa M, et al (2011) Acoustic radiation force impulse elastography as compared to transient elastography and liver biopsy in patients with chronic hepatopathies. Ultraschall Med 32: S46–52. 20603783 10.1055/s-0029-1245360

[23] de Lédinghen V, Vergniol J (2010) Transient elastography for the diagnosis of liver fibrosis. Expert Rev Med Devices 7: 811–823. 21050091 10.1586/erd.10.46

[24] Lindblad A, Glaumann H, Strandvik B (1999) Natural history of liver disease in cystic fibrosis. Hepatology 30: 1151–1158. 10534335 10.1002/hep.510300527

[25] Chen AH, Innis SM, Davidson AG, James SJ (2005) Phosphatidylcholine and lysophosphatidylcholine excretion is increased in children with cystic fibrosis and is associated with plasma homocysteine, S-adenosylhomocysteine, and S-adenosylmethionine. Am J Clin Nutr 81: 686–691. 15755840 10.1093/ajcn/81.3.686

[26] Nash KL, Allison ME, McKeon D, Lomas DJ, Haworth CS, et al (2008) A single centre experience of liver disease in adults with cystic fibrosis 1995–2006. J Cyst Fibros 7: 252–257. 18042441 10.1016/j.jcf.2007.10.004

[27] Gaia S, Carenzi S, Barilli AL, Bulgianesi E, Smedila A, et al (2011) Reliability of transient elastography for the detection of fibrosis in non-alcoholic fatty liver disease and chronic viral hepatitis. J Hepatol 54: 64–71. 20932598 10.1016/j.jhep.2010.06.022

[28] England K, Thorne C, Pembrey L, Tovo PA, Newell ML (2009) Age- and sex-related reference ranges of alanine aminotransferase levels in children: European paediatric HCV network. J Pediatr Gastroenterol Nutr 49: 71–77. 19465871 10.1097/MPG.0b013e31818fc63b

